# Corrections

**DOI:** 10.1016/S1473-3099(18)30412-2

**Published:** 2018-08

*Llanos-Cuentas A, Casapia M, Chuquiyauri R, et al. Antimalarial activity of single-dose DSM265, a novel Plasmodium dihydroorotate dehydrogenase inhibitor, in patients with uncomplicated* Plasmodium falciparum *or* Plasmodium vivax *malaria infection: a proof-of-concept, open-label, phase 2a study.* Lancet Infect Dis *2018; **18:** 874–83*—the open access license of this Article has been corrected to a Gold CC BY license. This correction has been made to the online version as of June 18, 2018, and will be made to the printed Article.

